# Steroid induced hypertriglyceridemia in pregnant waman with immune thrombocytopenia – case report

**DOI:** 10.1016/j.amsu.2022.103636

**Published:** 2022-04-15

**Authors:** Rehab Y. AL-Ansari, Faisal Ahmed Abu shaigah, Laila Alromaih, Moutaz Osman

**Affiliations:** a Adult Hematology Unit, Internal Medicine Department, KFMMC, Dhahran, 31932, Saudi Arabia; b Internal Medicine Department, KFMMC, Dhahran, 31932, Saudi Arabia; c Endocrine Unit, Internal Medicine Department, KFMMC, Dhahran, 31932, Saudi Arabia

**Keywords:** Steroid, Hypertriglyceridemia, Pregnant, Immune thrombocytopenia

## Abstract

**Background:**

Hypertriglyceridemia is a medical condition defined as fasting triglyceride level more than 150 mg/dl. It could be due to either familial or acquired cause as in obesity, metabolic syndrome, diabetes mellitus type 2, alcohol consumption, decrease exercise or drug affects. Drugs such as corticosteroids rarely induced hypertriglyceridemia, for that we are reporting this case.

**Case presentation:**

We are reporting a 35 years old pregnant lady diagnosed with immune thrombocytopenia and started on prednisolone 1mg/kg per oral once a day. Two months later, while on 20 mg of prednisolone, she presented to the emergency department with epigastric pain, nausea and vomiting for 15 days. Physical examination showed dry mucosa, new xanthelasma over both eyelids and epigastric tenderness with palpable suprapubic gravida uterus; otherwise, was unremarkable. Blood samples were highly lipemic, and laboratory investigations showed high triglycerides (TG) of greater than 73 mmol/L, mild diabetic keto acidosis with normal other chemistry including hepatic, renal, and pancreatic panel. She was treated by diet restriction, insulin infusion, Fenofibrate, and Omega 3 as well as rapid tapering down of prednisolone.

**Conclusion:**

Corticosteroid-induced hypertriglyceridemia is an uncommon condition and could be fatal, especially in high-risk cases. Unfortunately, no guidelines support a regular screening for lipid profile prior to initiating steroid therapy. However, we are suggesting a further study and creating a recommendation to mandate screening for lipid profile along with fasting blood sugar prior to initiating steroid therapy, especially in high-risk cases as in pregnancy.

## Highlights

• Screening for lipid profile along with fasting blood sugar prior to initiating steroid therapy, especially in high-risk cases as in pregnancy**.**
• Corticosteroid-induced hypertriglyceridemia is an uncommon condition and could be fatal.
• Patient with very high triglyceride level could present with abdominal pain, nausea and vomiting resampling pancreatitis but with normal amylase & lipase.

## 1 Introduction

Hypertriglyceridemia is associated with an increased risk of cardiovascular disease and pancreatitis (when the TG level is > 150 mg/dl and >500 mg/dl, respectively) [1]. Early detection of the disease and controlling the level is of great value and impact on patients' life. However, not only familial but also metabolic causes as well as some drugs can increase the risk of hypertriglyceridemia. Loop diuretics, Thiazide diuretics (high dose), Estrogen, Protease inhibitors, Cyclosporine, tacrolimus, First and second-generation antipsychotics and Retinoids, all are examples of drugs that could induce hypertriglyceridemia [2]. Chronic use of Corticosteroids, rather than acute or use for short duration, could predispose to hyperlipidemia or hypertriglyceridemia. However, there are conflicts about the degree of lipid profile affected by corticosteroid [3]. In a study by Yutaka MIBAYASHI, the effects of corticosteroid injection on triglyceride level among albino rates were measured, and he concluded that high TG level occurred as a result of increased free fatty acid as well as immunoreactive insulin and plasma glucose [4]. Furthermore, not much data or reports were found about corticosteroid-induced high TG level. As well as, no guideline about lipid profile screening prior to corticosteroid use, for that, we are reporting our case. This article has been reported in with the SCARE criteria [5].

## 2 Case report

### 2.1 History and examination

A 35-years-old pregnant female with a gestational age of 26 weeks, on prednisolone 20 mg once a day for the diagnosis of autoimmune thrombocytopenia in the past two months. Presented to the emergency department with worsening epigastric pain along with nausea and vomiting for 15 days. Epigastric pain was non-radiating and had no specific relieving or aggravating factors. There was no history of fever, change in level of consciousness, change in bowel habit, genitourinary symptoms, skin rash, ecchymosis, nor bleeding tendency.

Her past medical history was positive for single admission two months prior to her current presentation with bruises and epistaxis, was found to have a critical platelet count of 25 × 10^3^/μl. A diagnosis of autoimmune thrombocytopenia was established after extensive workup, ruling out connective tissue disease, infectious causes such as viruses and Helicobacter pylori, and hematology malignancy. Patient was initiated on prednisolone 1mg/kg/day with plan of tapering after response.

**Fig. 1:**
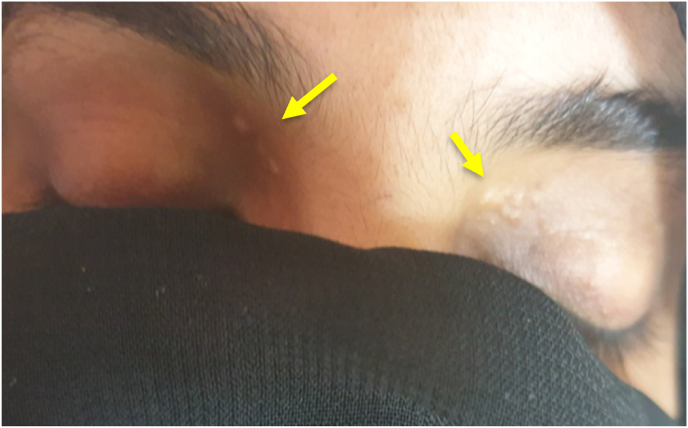
Xanthelasma over medial aspect of eye lids bilaterally (yellow arrows). (For interpretation of the references to colour in this figure legend, the reader is referred to the Web version of this article.)

On physical examination, her vital signs including blood pressure, pulse rate, temperature and oxygen saturation were normal with no orthostatic hypotension. She had dry oral mucosa and increased skin turgor on examination. There was new Xanthelasma over both eyelids bilaterally, which was not visible during previous admission (Fig. 1). Abdominal palpation revealed epigastric tenderness with palpable suprapubic gravida uterus; otherwise, the examination was not remarkable.

### 2.2 Laboratory result

**Fig. 2:**
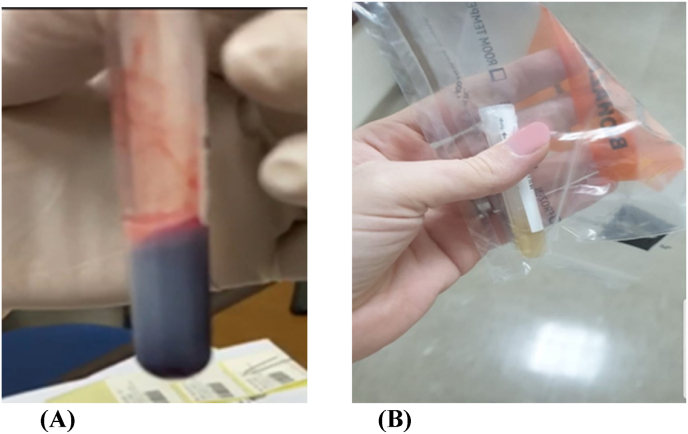
(**A**) Highly lipemic blood sample. (**B**) Stored sample from previous admission, not lipidemic.

Blood samples were highly lipemic and was difficult to run certain laboratory tests initially (Fig. 2). Hemoglobin was 12.7g/dl, white blood cells of 5.9 x10^3^/μl with normal differential and a platelet count of 100 x10^3^/μl. Inflammatory markers, renal function, liver enzymes as well as amylase lipase were within normal.

Coagulation profile showed prolonged PT and normal APTT 170 and 25 sec respectively, low fibrinogen of less than 0.6g/L and high INR of more than 16 on initial lipemic sample. The second sample mixing study showed all within normal results of PT and APTT 10 and 22 seconds respectively, INR of 0.91 and fibrinogen of 3.27g/L.

Lipid profile showed triglycerides of greater than 73 mmol/L and cholesterol of 14.6 mmol/L, ALDL and HDL were within normal limits. Venous blood gas sample was consistent with metabolic acidosis and respiratory alkalosis with an anion gap of 16 and a urine dipstick was significant for +2 ketones. Random blood sugar was14 mmol/L. Ultrasound abdomen showed mild fatty and enlarged liver with homogenous bright echo-pattern with multiple gallstones.

Stored blood product sample from previous admission was not lipidemic.

### 2.3 Hospital course and follow up

Initially and at the point of presentation we were considering the differential diagnosis of acute abdomen as in acute pancreatitis, acute cholecystitis, cholangitis, peptic ulcer disease and less likely mesenteric ischemia, others as late onset hyperemesis gravidarum was also considered. However, after initial laboratory and radiological study results, our initial differential diagnosis were excluded.

The patient was admitted to critical care area for observation with a diagnosis of hypertriglyceridemia and mild diabetic ketoacidosis secondary to corticosteroid use. She received insulin infusion, Fenofibrate, and Omega 3. Dietary restriction to low fat, low carbohydrates and high Omiga-3-faty acid and proteins were implemented. Prednisolone was rapidly tapered down and replaced by two doses of intravenous immunoglobulin during her hospital stay for rapid dropping in her platelet count after holding corticosteroids. Upon discharge from hospital, TG level was 3 mmol/L, platelets of 130 x10^3^/μl and a normal random blood sugar.

The patient continued to follow up at the outpatient department, where prednisolone was tapered to 10mg until admitted for delivery at 38 weeks of gestation, with platelet level maintained above 80 x10^3^/μl and TG level reached 2.35 mmol/L. Post-delivery, which was normal vaginal delivery, prednisolone was tapered further to 5mg after platelet level was stable above 100 x10^3^/μl with a future plan of discontinuing medication; unfortunately, the patient lost follow-up.

## 3 Discussion

Immune thrombocytopenia (ITP) is a condition where platelet count goes below 100 × 10⁹/L level with no clear other causes for low platelet count as viral illness, drug, connective tissue disease, hematological malignancy, or familial cause. It is a disease of exclusion with no definitive diagnostic or prognostic markers. Moreover, not all cases require treatment, most cases stay under observation as far as platelet level more than 30 × 10⁹/L and no bleeding. Corticosteroid is the first and frontline management for active immune thrombocytopenia once required with an 80% response rate. Other modality of treatment includes intra venous immunoglobulins (IVIG), Anti-Rh(D), Rituximab, Thrombopoietin receptor agonists, Cyclophosphamide, Cyclosporine, Danazol, Dapsone, and Splenectomy [6].

ITP affects about 3–4% of pregnant ladies presenting with thrombocytopenia, especially in the first trimester [7]. Similar to the non-pregnant population, the goal is to maintain the patient at safe level of platelet around 30 × 10⁹/L with no bleeding, especially in the first or second trimester. On the other hand, some recommend a platelet count of around 50 × 10⁹/L at the third trimester or >70 × 10⁹/L if epidural anesthesia required [7,8]. However, corticosteroid could increase the risk of gestation diabetes, hypertension, placenta previa and abruption of the placenta. For such complications, some prefer starting IVIG or anti-Rh(D) instead of corticosteroid but no strong recommendations or guidelines [[7], [8], [9], [10]]. In our case scenario, she was started on corticosteroid as her platelet count was down to 25 × 10⁹/L with active epistaxis and bruises during previous admission after being investigated for other causes of thrombocytopenia. Furthermore, blood glucose level and blood pressure were assessed prior to initiation of corticosteroid and during follow-up.

The effect of long-term corticosteroids for controlling connective tissue disease and bronchial asthma on lipid profile was reported by Jefferys DB et al., which concluded that corticosteroids might elevate the total cholesterol and high-density lipoprotein level notably in the female gender [11]. On the other hand, a study of the effects of corticosteroids among US population that was run by Choi HK et al. showed no association between corticosteroids and high TG or total cholesterol levels [12]. However, other studies reported high TG levels rather than total cholesterol levels in bronchial asthma as well as in Systemic Lupus Erythematosus patients on long-term corticosteroid therapy [13,14]. There are two mechanisms behind the effect of corticosteroids on lipid profiles. Either due to facilitation effects of lipolytic agents or due to redistribution of the fat in the upper parts of the body, as in trunk and face, this will affect the distribution of glucocorticoid's receptors in the fat cells. Therefore, when there is a high number of receptors, this will decrease glucose uptake and reduce TG accumulation [15]. Nonetheless, no report or study was found about corticosteroid-induced high TG level in short-time or acute use such in our case scenario.

Management of high triglyceride levels includes dietary control by lowering carbohydrates with increasing protein and fat as omega-3 fatty acids as well as moderate to high-intensity exercise.

The role of medications as Statins can be considered for patients with mild or intermediate risk of atherosclerotic cardiovascular disease. On the other hand, patients at high risk for cardiovascular mortality may consider using high-dose icosapent. However, for very high TG levels, fibrates and omega-3 fatty acids can be used to reduce the risk of pancreatitis. Insulin infusion should be considered in case of hypertriglyceridemia induced acute pancreatitis. Whenever triglyceride levels >1000 mg/dL, this will indicate plasmapheresis [1].

## 4 Conclusion

Corticosteroid-induced hypertriglyceridemia is an uncommon condition and could be fatal, especially in high-risk cases. Unfortunately, no guidelines support a regular screening for lipid profile prior to initiating steroid therapy. However, we are suggesting a further study and creating a recommendation to mandate screening for lipid profile along with fasting blood sugar prior to initiating steroid therapy, especially in high-risk cases such as pregnancy, family history for hyperlipidemia or hypertriglyceridemia, obese, smoker, diabetic or cardiovascular patients.

## Ethical approval

Ethical approval not required for case report as per our institute ethical and research committee.

## Sources of funding

No found was provided.

## Author contribution

Dr rehab = wrote manuscript.

Dr faisal = wrote case report.

Dr Laila = wrote abstract.

Dr Osman = reviewer.

## Consent

Written informed consent was obtained from the patient for publication of this case report and accompanying images.

## Registration of research studies

1. Name of the registry:

Not applicable.

2. Unique Identifying number or registration ID:

Not applicable.

3. Hyperlink to your specific registration (must be publicly accessible and will be checked):

Not applicable.

## Guarantor

Dr Rehab Al-Ansari.

## Trial registry number

Not applicable.

## Provenance and peer review

Not commissioned, externally peer reviewed.

## Declaration of competing interest

No conflict of interest.
